# Testicular toxicity of Aroclor 1254 in selenium-deficient and selenium-supplemented rats

**DOI:** 10.2478/aiht-2026-77-4027

**Published:** 2026-06-30

**Authors:** Pınar Erkekoğlu, Aylin Balcı-Özyurt, Naciye Dilara Zeybek, Hülya Tezel-Yalçın, Ünzile Yaman, Gizem Yildiztekin, Sevtap Aydın-Dilsiz, Murat Kızılgün, Evin İşcan, Tuğçe Batur, Mehmet Öztürk, Oğuz Han Edebal, Nurşen Başaran, Belma Koçer-Gumusel

**Affiliations:** Hacettepe University Faculty of Pharmacy, Department of Pharmaceutical Toxicology, Sıhhiye, Ankara, Turkey; Bahçeşehir University Faculty of Pharmacy, Department of Pharmaceutical Toxicology, Istanbul, Turkey; Hacettepe University Faculty of Medicine, Department of Histology and Embryology, Sıhhiye, Ankara, Turkey; İzmir Katip Çelebi University Faculty of Pharmacy, Department of Pharmaceutical Toxicology, Izmir, Turkey; Erzincan Binali Yıldırım University Faculty of Pharmacy, Department of Pharmaceutical Toxicology, Erzincan, Turkey; University of Health Sciences, Gülhane Faculty of Medicine, Ankara, Turkey; Dokuz Eylul University, Izmir Biomedicine and Genome Centre, Izmir, Turkey; Izmir Tinaztepe University Galen Research Centre, Izmir, Turkey; Near East University Hospital, Department of Biochemistry, Nicosia, Cyprus; Başkent University Faculty of Pharmacy, Department of Pharmaceutical Toxicology, Ankara, Turkey; Okan University Faculty of Pharmacy, Department of Pharmaceutical Toxicology, Istanbul, Turkey

**Keywords:** apoptosis, oxidative stress, polychlorinated biphenyls, Se, sperm, apoptoza, oksidacijski stres, poliklorirani bifenili, selen, spermiji

## Abstract

Aroclor 1254 (A1254) is a mixture of polychlorinated biphenyl (PCB) congeners known to induce testicular toxicity through oxidative stress, apoptosis, and hormonal disruption. Selenium (Se), an essential trace element for male reproduction, contributes to antioxidant defence, DNA repair, and spermatogenesis. Given the worldwide prevalence of Se deficiency and its potential impact on reproductive health, this study investigated the effects of A1254 exposure on testicular function in Sprague-Dawley rats with different Se status. Rats assigned to the Se-deficient group were receiving a ≤0.05 mg/kg Se diet, while those assigned to the Se-supplemented group were receiving 1 mg/kg Se for five weeks. A1254-treated groups were receiving 10 mg/kg/day of A1254 by oral gavage for 15 consecutive days. A1254 exposure caused significant reductions in testis weight, sperm count, motility, and plasma testosterone levels, accompanied by extensive germ cell apoptosis and enhanced lipid and protein oxidation. Total antioxidant capacity also dropped significantly, confirming redox imbalance in A1254-exposed testes. These adverse effects were substantially exacerbated in the Se-deficient group, whereas Se supplementation ameliorated but did not fully prevent histological and biochemical changes induced by A1254. Our findings demonstrate that Se plays a crucial protective role against PCB-associated testicular injury, primarily by mitigating oxidative stress and apoptotic pathways. However, Se supplementation alone may not completely counteract the reproductive toxicity caused by persistent environmental contaminants such as A1254.

## Abstract (hr)

Aroclor 1254 (A1254) mješavina je kongenera polikloriranih bifenila (PCB), za koje je poznato da prouzročuju toksičnost u testisima putem oksidacijskoga stresa, apoptoze i hormonskih poremećaja. Selen (Se), esencijalni element u tragovima važan za mušku reprodukciju, pridonosi antioksidacijskoj obrani, popravku DNA i spermatogenezi. S obzirom na raširenu pojavnost nedostatka selena u svijetu i njegov mogući utjecaj na reproduktivno zdravlje, u ovom su istraživanju ispitani učinci izloženosti A1254 na funkciju testisa u Sprague-Dawley štakora s različitim statusom selena. Štakori u skupini s manjkom selena primali su ga u hrani u masenoj frakciji ≤0,05 mg/kg, dok su životinje u nadomjesnoj skupini primale 1 mg/kg selena tijekom pet tjedana. Skupine tretirane A1254 primale su ga 10 mg/kg/dan oralnom gavažom tijekom 15 uzastopnih dana. Izloženost A1254 prouzročila je značajno smanjenje mase testisa, broja i pokretljivosti spermija te razine testosterona u plazmi, uz zamjetnu apoptozu zametnih stanica i pojačanu oksidaciju lipida i proteina. Ukupni antioksidacijski kapacitet također se značajno smanjio, što potvrđuje narušenu redoks ravnotežu u testisima izloženima A1254. Ovi štetni učinci bili su znatno izraženiji u skupini s manjkom Se, dok je nadomjestak ublažio, ali nije potpuno spriječio histološke i biokemijske promjene izazvane A1254. Naši rezultati pokazuju da selen ima ključnu zaštitnu ulogu protiv oštećenja testisa povezanih s PCB-ima, ponajviše ublažavanjem oksidacijskoga stresa i inhibicijom apoptotskih putova. Međutim, sama suplementacija selenom možda nije dostatna za potpuno suzbijanje reproduktivne toksičnosti prouzročene postojanim okolišnim onečišćivalima poput A1254.

Polychlorinated biphenyls (PCBs) are a class of organic chlorine compounds widely used in various industrial applications, such as transformers, capacitors, and plastics, due to their dielectric properties (1,2,3). Commercial PCB mixtures are known as Aroclors, and Aroclor 1254 (A1254) is a particularly widespread member of this family (4, 5). Despite banned production since 1979 due to increasing evidence of their toxicity (6), PCBs persist in soil, air, and water (1, 7) and bioaccumulate further down the food chain, which is the primary route of human exposure (7, 8). Being poorly metabolised, PCBs persist in various tissues, including skin, adipose tissue, and the testes (9), where they interact with oestrogen and androgen receptors (10).

A1254 has been implicated in numerous adverse effects on male reproductive health in both animals and humans (7, 11). The primary mechanism of A1254 toxicity involves activating the aryl hydrocarbon receptor (AhR) and profoundly disrupting the prooxidant-antioxidant balance within the testes (5, 12). This imbalance leads to the excessive generation of reactive oxygen species (ROS), resulting in cellular dysfunction, elevated lipid peroxidation, protein oxidation, and reduced glutathione levels (11, 13). Consequently, A1254 exposure can cause severe damage, including lower testosterone levels, reduced sperm motility and count, DNA damage, infertility, and testicular atrophy (1, 2, 5, 7, 12,13,14). Given that this ROS-dependent mechanism is central to testicular dysfunction, maintaining testicular antioxidant/oxidant balance is crucial for the proper functioning of Leydig, Sertoli, and sperm cells (2, 7, 10,11,12, 15).

This redox balance is naturally maintained by selenium (Se) as a component of selenoproteins and major antioxidant enzymes like glutathione peroxidase (GPx) (16,17,18). Its deficiency is linked to various reproductive disorders (4, 18), yet, to the best of our knowledge, no studies in the literature have specifically addressed the protective role of Se against A1254-induced testicular toxicity.

The aim of our study was to address this gap by determining whether Se deficiency or supplementation could modulate the toxic effects of A1254 on rat testicular histology, oxidative stress biomarkers, sperm parameters, germ cell apoptosis, plasma testosterone and oestradiol levels, and cell cycle checkpoint proteins.

## MATERIALS AND METHODS

### Chemicals and kits

A1254 (CAS No.11097-69-1), formalin solution (CAS No. 50-00-0), ethyl alcohol (CAS No. 64-17-5), paraffin (CAS No. 8042-47-5) Tris (CAS No. 77-86-1), diethylenetriaminepentaacetic acid (CAS No. 67-43-6), phenylmethanesulfonyl fluoride (CAS No. 329-98-6), thiobarbituric acid (CAS No. 504-17-6), 1,1,3,3 tetraethoxypropane (CAS No. 102-52-3), glutathione reductase (CAS No. 9001-48-3), reduced glutathione (CAS No. 70-18-8), nicotinamide adenine nucleotide phosphate (CAS No. 53-59-8), methanol (CAS No. 67-56-1), potassium dihydrogen phosphate (CAS No. 7778-77-0), potassium hydroxide (CAS No. 1310-58-3), protease inhibitor cocktail and bovine serum albumin (BSA) (CAS No. 9048-46-8) were obtained from Sigma-Aldrich (St. Louis, MO, USA). Merck Masson’s trichrome (CAS No. 12026-57-2), Mayer’s haematoxylin solution, phosphate-buffered saline (PBS), Periodic Acid-Schiff (PAS) kit, hydrogen peroxide (CAS No. 7722-84-1), pyrogallol (CAS No. 87-66-1), and *n*-butanol (CAS No. 71-36-3) were purchased from Merck (Darmstadt, Germany). Terminal deoxynucleotidyl transferase-mediated deoxyuridine triphosphate nick end-labelling (TUNEL) Cell Death Detection kit was purchased from Roche (Mannheim, Germany).

Total antioxidant capacity (TAOC, Cat No. 709001), thiobarbituric acid reactive substances (TBARS, Cat No. 10009055), protein carbonyl (Cat No. 10005020), glutathione peroxidase (GPx1, Cat No. 703102), catalase (CAT, Cat No. 707002), and superoxide dismutase (SOD, Cat No. 706002) assay kits were obtained from Cayman Chemical Company (Ann Arbor, MI, USA). Selenoprotein P (SePP) kit was obtained from East Biopharm Company (Hangzhou, China). Thioredoxin reductase (TrxR, Cat No. CS0170-1KT) and glutathione (GSH, Cat No. MAK517-1KT) assay kits were purchased from Sigma-Aldrich. The caspase-3 activity (Cat No. KSA626Hu01) and the caspase-8 activity (Cat No. SEA853Hu) kits were purchased from Cloud-Clone Corp. (Katy, TX, USA).

Animal feed was supplied from Scientific Animal Food and Engineering (SAFE) Laboratories (Augy, France). Bicinchoninic acid (BCA) protein assay kit was from Thermo Fisher Scientific (Waltham, MA, USA). Rat testosterone enzyme-linked immunosorbent assay (ELISA) kit (Cat No. MBS702057) and rat oestradiol ELISA kits (Cat No. MBS702969) were obtained from MyBioSource (San Diego, CA, USA).

PureSperm Wash was from Nidacon (Mölndal, Sweeden), and the DiffQuik Stat III Sperm Staining kit from Electron Microscopy Sciences (Hatfield, PA, USA). All other chemicals were purchased from Merck.

### Animal and treatment

All experimental procedures were approved by the Hacettepe University Animal Ethics Committee (approval No. ET-15.066) and conducted in accordance with the guidelines of the Turkish Ministry of Food, Agriculture and Livestock, the National Institutes of Health, and the National Centre for the Replacement, Refinement and Reduction of Animals in Research.

The study involved 36 three weeks old male Sprague-Dawley rats. We did not run power analysis to determine the sample size but followed the maximum permissible limit set by the Institutional Animal Ethics Committee. The animals were kept under controlled temperature (23 °C), humidity (50 %), and a 12:12 h light-dark cycle and had free access to food and water.

They were randomised into six experimental groups (n=6 per group): control (receiving 0.15 mg/kg of Se for five weeks and 1 mL of corn oil for the last 15 days of the five-week experimental period), Se-supplemented (receiving 1 mg/kg of Se per day for five weeks; SeS), Se-deficient (receiving 0.05 mg/kg of Se per day for five weeks; SeD), Aroclor 1254 (receiving 0.15 mg/kg/day of Se for five weeks and 10 mg/kg/day of A1254 in corn oil for the last 15 days of the experiment; A1254), Aroclor 1254+Se-supplemented (ASeS), and Aroclor 1254+Se-deficient (ASeD).

Selenium and Aroclor 1254 doses were selected based on previously published protocols (15,16,17,18,19). After the five-week experimental period, the rats were euthanised under thiopental anaesthesia, and their testes excised and weighed.

Blood samples were collected into heparinised tubes after euthanasia and the plasma separated by centrifugation at 800 *g* for 15 min. Supernatants were collected and kept at −80 °C until testosterone and oestradiol measurement.

Left testis of each animal was immediately frozen in liquid nitrogen and kept at −80 °C. The next day, testis homogenates were prepared in ice-cold buffer containing Tris (10 mmol/L; adjusted to pH 7.4) and protease inhibitors [diethylenetriamine pentaacetic acid (DTPA, 1 mmol/L), and phenylmethanesulphonyl fluoride (PMSF, 1 mmol/L)] using a Teflon pestle homogeniser to obtain the whole 10 % (w/v) homogenate. After centrifugation at 1500 *g* and 4 °C for 10 min, TAOC, malondialdehyde (MDA), and carbonyl group concentrations were measured in half of the supernatant. The other half was re-centrifuged at 4000 *g* and 4 °C for 10 min to measure antioxidant enzyme activities and GSH levels.

### Histopathological evaluation

For histopathology we used samples taken from the right testis fixed rapidly in 10 % formalin, dehydrated through graded alcohols, and processed for routine light microscopy. All specimens were embedded in paraffin, and 6 µm sections cut and stained with haematoxylin and eosin (H&E) and Masson’s trichrome.

### TUNEL assay

The TUNEL assay was used to label apoptosis using a cell death detection kit according to the manufacturer’s instructions. Briefly, 5 µm thick testis sections fixed in Bouin and embedded in paraffin were mounted on adhesive pre-treated glass slides, deparaffinised, rehydrated, and washed twice in PBS for 5 min. Then, the slides were incubated with a permeabilisation solution of 0.1 % Triton X-100 in 0.1 % sodium citrate at 4 °C for 8 min and washed twice with PBS for 5 min, The labelling reaction was performed using 50 µL of the TUNEL reagent for each sample, save for the negative control (which was added the reagent without enzyme and incubated at 37 °C for 1 h). Following another round of PBS washing, the slides were incubated with the converter reagent at 37 °C for 30 min. After washing, cells containing labelled DNA strand breaks were stained by incubating the slides with 3,3’-diaminobenzidine (DAB) solution for 10 min. TUNEL-positive cells were counted in 20 seminiferous tubules for each slide. All slides were examined under a Leica DM6000B microscope and photographed with a Leica DC490 digital camera (Leica Microsystems GmbH, Wetzlar, Germany).

### Sperm parameter measurements

After right testis removal, the right epididymis was trimmed and separated into the caput and cauda (i.e. the caput and corpus were separated at the neck, whereas the corpus and cauda were separated at the site where engorged tubules were first recognised). We then recorded the weight of the cauda, placed it into a glass Petri dish containing PureSperm Wash supplemented with 0.5 % BSA, and minced with anatomic scissors. The suspension was centrifuged at 800 *g* for 10 min and the pellet diluted with PureSperm Wash. Then, 10 µL of the suspension was applied to Neuber haemocytometer for sperm count and motility, and 100 sperms were assessed by manual counting for progressive sperm motility under a microscope (Leica) at 200× magnification.

The same diluted samples were also used for sperm morphology. First, 10 µL of the suspension was applied to a slide and stained with the DiffQuik Stat III Sperm Staining kit. Abnormal and normal sperms were counted manually (100 per slide, counted in duplicate) at 400× magnification, and abnormal sperms classified as follows: tail without head, head without tail, big head, small head, amorphous head, other head abnormalities, mid-piece anomaly, and tail anomaly, as described elsewhere (20).

### Sex hormone measurements

Rat testosterone was measured with the quantitative competitive ELISA kit, whose detection range is 0.125–25 ng/mL and sensitivity 0.04 ng/mL. The microtitre plate provided in this kit was pre-coated with goat-anti-rabbit antibody. Standards or samples were added to the appropriate microtitre plate wells with an antibody specific for testosterone and horseradish peroxidase (HRP) conjugated testosterone. The competitive inhibition reaction was launched between HRP-labelled and unlabelled testosterone. A substrate solution was added to the wells, stained, and colour intensity measured at 450 nm using the SpectraMax M2 microplate reader (Molecular Devices, San Jose, CA).

Rat oestradiol was measured with the quantitative double antibody sandwich ELISA kit, whose detection range is 15.6–1000 pg/mL and sensitivity 1 pg/mL. It is pre-coated with an anti-rat E2 monoclonal antibody. Briefly, plasma samples and biotinylated polyclonal antibodies for detection were added into wells and washed with PBS. Next, avidin-peroxidase conjugates were added, and unbound conjugates thoroughly washed out of the wells with PBS. The 3,3′,5,5′-tetramethylbenzidine (TMB) was then used to stain the substrate. TMB that reacts with peroxidase turns blue and then yellow, after the addition of the stop solution. The absorbance was measured at 450 nm, as colour intensity corresponds to the quantity of the target analyte.

### Determination of antioxidant enzyme activities and oxidative stress parameters

Glutathione peroxidase 1 (GPx1), catalase (CAT), superoxide dismutase (SOD), and thioredoxin reductase (TrxR) activities were determined with commercial kits. GPx1 activity was determined by coupling the reduction of t-butyl hydroperoxide to NADPH oxidation via glutathione reductase (GR) to maintain constant reduced glutathione (GSH) levels. The continuous decrease in NADPH absorbance was monitored spectrophotometrically at 340 nm, where one unit (U) of GPx1 was defined as the amount of enzyme transforming 1 μmol of NADPH to NADP^+^ per minute at 37 °C, expressed as U/mg protein.

CAT activity was assessed by stopping the enzymatic decomposition of H_2_O_2_ and detecting the residual substrate at 520 nm using a chromogen solution (150 mmol/L potassium phosphate buffer, pH 7.0, containing 0.25 mmol/L 4-aminoantipyrine and 2 mmol/L 3,5-dichloro-2-hydroxybenzenesulfonic acid). One unit of CAT was defined as the amount of enzyme decomposing 1 mmol/L of H_2_O_2_ per minute at pH 7.0 and 25 °C, expressed as nmol/min/mg protein.

SOD activity was determined colourimetrically utilising the cell proliferation reagent WST-1, which forms a water-soluble formazan dye upon reduction by superoxide anions generated by xanthine oxidase (XO). SOD-mediated inhibition of this reduction was quantified by tracking the decrease in absorbance at 440 nm to calculate the IC_50_ value, with activity expressed as U/mg protein.

TrxR activity was determined colourimetrically using the thioredoxin reductase assay kit. The method is based on the reduction of 5,5′-dithiobis-(2-nitrobenzoic) acid (DTNB) with NADPH into 5-thio-2-nitrobenzoic acid (TNB), the concentration of which was measured at 412 nm. It is expressed in units (U), where one unit corresponds to the amount of enzyme causing an increase of 1.0 in absorbance per minute at 412 nm in 1 mL of reaction mixture under the assay conditions (pH 7.0, 25 °C).

TAOC, GSH, lipid peroxidation, and protein oxidation were determined using commercial kits. The capacity of the antioxidants in the sample to prevent ABTS^+^ oxidation was measured spectrophotometrically at 405 nm and quantified in mmol/L Trolox equivalents.

Total GSH content was assessed using a kinetic assay in which GSH reduces 5,5′-dithiobis-(2-nitrobenzoic) acid (DTNB) to TNB, measured spectrophotometrically at 412 nm (21) and quantified against a standard curve of known total GSH concentrations. The results are expressed in nmol/mg protein.

Plasma lipid peroxidation levels were quantified using a TBARS assay kit, which involves the spectrophotometry measurement of the MDA complex with thiobarbituric acid (TBA) under high temperature (90–100 °C) and acidic conditions at 540 nm. The amount of MDA in the samples was calculated against MDA standards (0, 0,5, 5, 10, 20, 30, and 50 µmol/L), and results expressed as µmol/L.

Protein oxidation was established by measuring spectrophotometrically the formation of stable dinitrophenyl (DNP) hydrazone adducts at 360 nm, which is proportional to the amount of carbonyls present in the samples.

SePP was determined using the double-antibody sandwich ELISA kit. SePP interacts with monoclonal antibody in the pre-coated wells to form an immune complex, whose concentration is measured spectrophotometrically at 450 nm by adding chromogen and corresponds to SePP concentration in the samples. The results are expressed as ng/µg protein.

All assays were performed in triplicate, and the coefficient of variation for all enzymatic assays was maintained below 10 %.

### Total protein determination

Protein contents of the testis homogenates were determined using the above mentioned BCA protein assay kit.

### Determination of caspase-3 and caspase-8 activities

The caspase-3 activity in testis homogenates was determined with the caspase-3 colourimetric assay kit based on the hydrolysis of acetyl-Asp-Glu-Val-Asp p-nitroanilide (Ac-DEVD-pNA) by caspase-3, resulting in the release of the *p*-nitroaniline (pNA) moiety, whose concentration was calculated from absorbance at 405 nm and corresponds to caspase-3 activity expressed as nmol/p-NA/min/mg protein. Similarly, caspase-8 activity is derived from pNA concentration calculated from the absorbance values at 405 nm and expressed as µmol/p-NA/min/mg protein. All samples were analysed in triplicate.

### Determination of p53 and p21 protein expressions

Protein expressions of p53 and p21 in tissues were analysed by Western blotting in triplicate, using beta-actin as a loading control and HepG2 cells as positive control. Tissues were pulverised in liquid nitrogen and lysed with the radioimmunoprecipitation assay (RIPA), while HepG2 cells were processed in a Triton X-100-based lysis buffer, both supplemented with protease and phosphatase inhibitors. After quantification via the BCA assay, 30 g of denatured protein per sample was resolved on SDS-PAGE gels (10 % for p53, 15 % for p21, with a 5 % stacking gel) using standard Tris-glycine running buffer. Proteins were transferred onto membranes, blocked with 5 % BSA in 0.5 % TBS-T, and probed with primary antibodies against p53 (1:1000), p21 (1:1000), and beta-actin (1:5000). Following incubation with appropriate anti-mouse or anti-rabbit secondary antibodies (1:2500), protein bands were visualised using chemiluminescence imaging.

### Statistical analysis

The results are expressed as means ± standard errors of the mean (SEM). As the Shapiro-Wilk test showed no normal distribution, the differences between the groups were evaluated with the Kruskal-Wallis one-way analysis of variance (ANOVA). The Mann-Whitney *U*-test was used for subsequent multiple comparisons corrected with the Bonferroni method. All analyses were run on Statistical Package for Social Sciences (SPSS) version 17.0 (Armonk, NY, USA), and statistical significance set to p<0.05.

## RESULTS

### Animal body weight gain and testis weights

**Figure 1:**
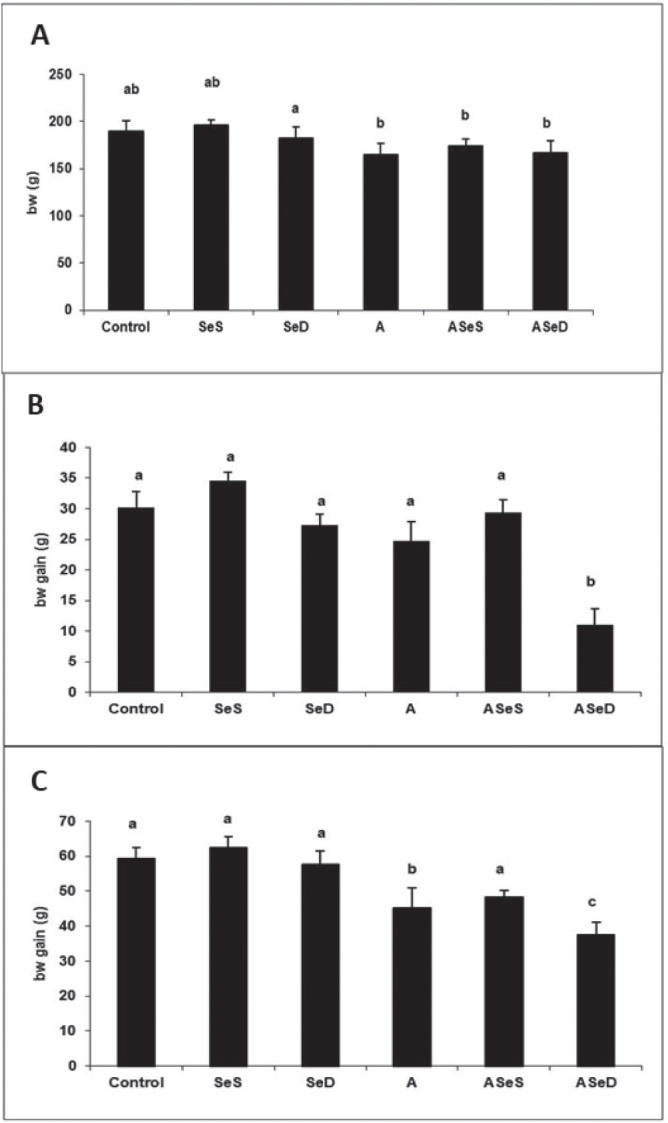
Mean (±SD) body weights (A) and body weight gain in the first week (B) and the last 14 days (C) of treatment. ^a, b^ Bars that do not share same superscript letters are significantly different (p<0.05). A – Aroclor 1254 group receiving 0.15 mg/kg/day of Se for 5 weeks and 10 mg/kg/day of Aroclor 1254 in corn oil for the last 15 days; ASeD – Se-deficient group receiving Aroclor 1254 as described above.; ASeS –Se-supplemented group receiving Aroclor 1254 as described above; Control – group receiving 0.15 mg/kg/day of Se for 5 weeks; SeD – Se-deficient group receiving 0.50 mg/kg/day of Se for 5 weeks; SeS – Se-supplemented group receiving 1 mg selenium/kg/day for 5 weeks

Figure 1 shows that body weight gain was significantly lower in the ASeD group compared to all other groups in the first week of A1254 treatment (p<0.05). In the second week, body weight gain was significantly lower in the A1254 group than in the control, SeS, SeD, and ASeS groups, while the ASeD group exhibited the lowest body weight gain of all groups (p<0.05). By the end of the experiment, final body weight did not differ significantly between the control and treatment groups, but the A1254, ASeS, and ASeD groups had significantly lower body weights than the SeD group (p<0.05).

**Figure 2:**
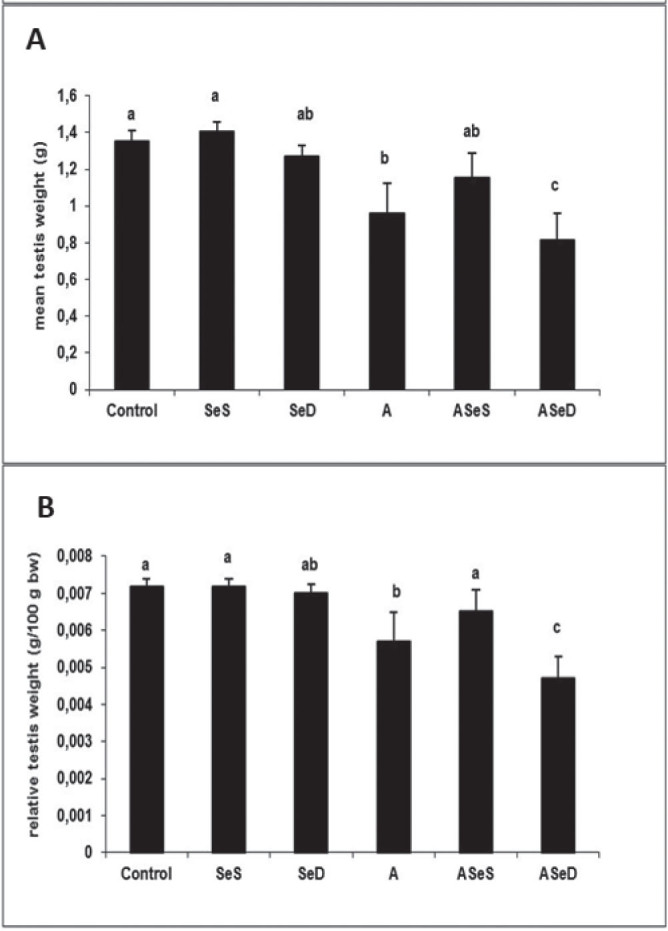
Mean (A) and relative (B) testis weights in our study groups. a, b, c Bars that do not share same superscript letters are significantly different (p<0.05). A – Aroclor 1254 group receiving 0.15 mg/kg/day of Se for 5 weeks and 10 mg/kg/day of Aroclor 1254 in corn oil for the last 15 days; ASeD – Se-deficient group receiving Aroclor 1254 as described above.; ASeS –Se-supplemented group receiving Aroclor 1254 as described above; Control – group receiving 0.15 mg/kg/day of Se for 5 weeks; SeD – Se-deficient group receiving 0.50 mg/kg/day of Se for 5 weeks; SeS – Se-supplemented group receiving 1 mg selenium/kg/day for 5 weeks

Figure 2 shows significantly lower mean and relative testis weights (respective to 100 g of body weight) in the A1254 and ASeD groups compared to control (p<0.05).

### Histopathological and TUNEL findings

**Figure 3:**
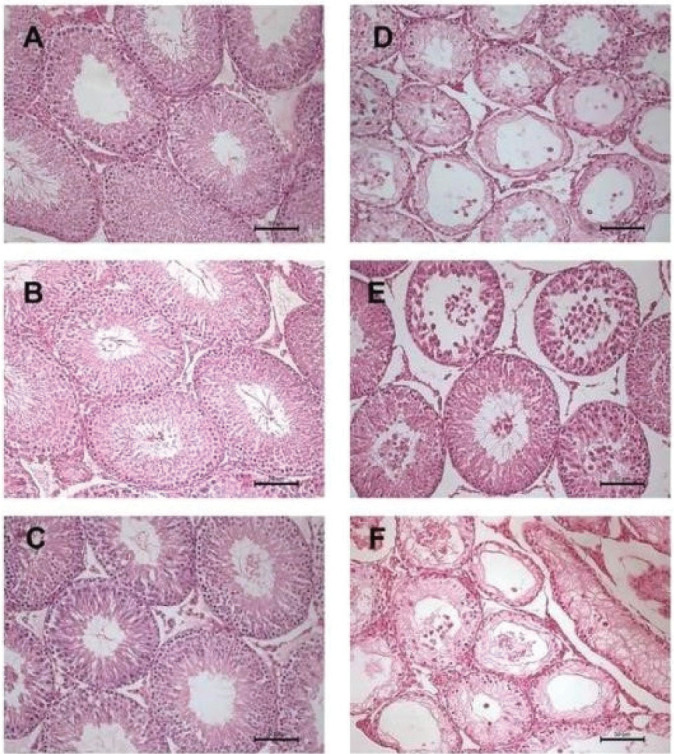
Histopathological changes in the study groups: seminiferous tubules with normal morphology in control (A), SeS (B), SeD (C) and ASeS (E) groups; prominent degeneration of seminiferous epithelium in A1254 (D) and ASeD groups (F) (haematoxylin-eosin, 200× magnification). A1254 – Aroclor 1254 group receiving 0.15 mg/kg/day of Se for 5 weeks and 10 mg/kg/day of Aroclor 1254 in corn oil for the last 15 days; ASeD – Se-deficient group receiving Aroclor 1254 as described above.; ASeS –Se-supplemented group receiving Aroclor 1254 as described above; Control – group receiving 0.15 mg/kg/day of Se for 5 weeks; SeD – Se-deficient group receiving 0.50 mg/kg/day of Se for 5 weeks; SeS – Se-supplemented group receiving 1 mg selenium/kg/day for 5 weeks

In line with these findings, Figure 3 shows prominent degeneration of the seminiferous epithelium in the A and ASeD groups (Figure 3D and 3F, respectively). The observed changes included degeneration, epithelial disorganisation, significant loss of germ cells, interstitial oedema and congestion. Sertoli cells were present, but neither spermatogenesis nor spermiogenesis was observed (Figure 3F). In the ASeS group, histological damage was mitigated, but some tubules showed desquamated cells (Figure 3E).

**Figure 4:**
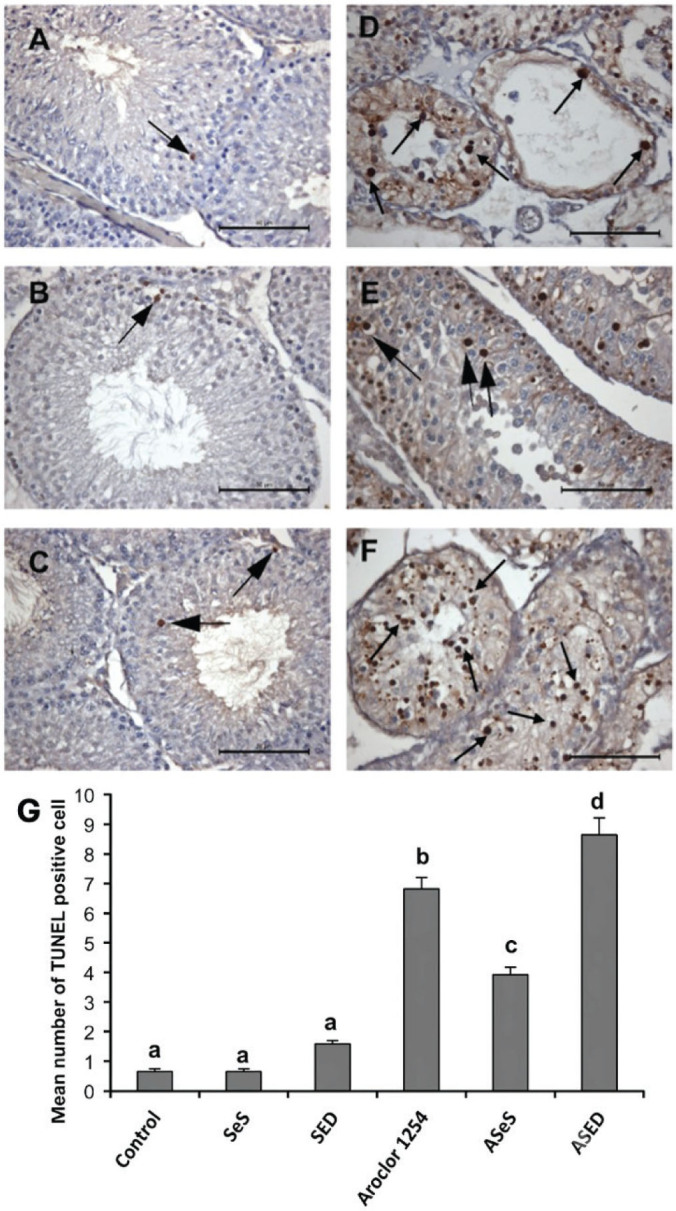
TUNEL-positive (apoptotic) cells in seminiferous tubules of A) control; B) SeS; C) SeD; D) A1254; E) ASeS; and F) ASED group indicated by arrows (400× magnification) and G) mean (±SD) apoptotic cell counts across the groups. ^a, b, c, d^ Bars that do not share same superscript letters are significantly different (p<0.05). A – Aroclor 1254 group receiving 0.15 mg/kg/day of Se for 5 weeks and 10 mg/kg/day of Aroclor 1254 in corn oil for the last 15 days; ASeD – Se-deficient group receiving Aroclor 1254 as described above.; ASeS – Se-supplemented group receiving Aroclor 1254 as described above; Control – group receiving 0.15 mg/kg/day of Se for 5 weeks; SeD – Se-deficient group receiving 0.50 mg/kg/day of Se for 5 weeks; SeS – Se-supplemented group receiving 1 mg selenium/kg/day for 5 weeks

Figure 4 shows apoptosis progression in germ cells revealed with the TUNEL assay. Se deficiency caused a significant, two-fold increase in apoptotic germ cells, and A1254 exposure approximately 11-fold increase compared to control (p<0.05). In the ASeS group, this number halved compared to the A1254 group (p<0.05) but increased 14 times in the ASeD group compared to control (p<0.05) and a 1.2 times compared to the A1254 group (p<0.05).

### Sperm parameters

**Figure 5:**
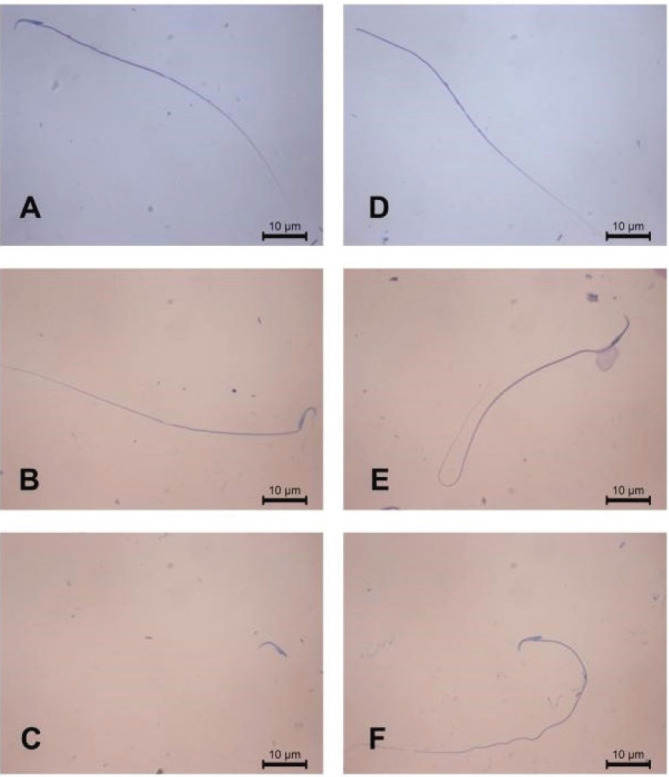
Sperm images across the A) control; B) SeS; C) SeD; D) A1254; E) ASeS; and F) ASeD group. A – Aroclor 1254 group receiving 0.15 mg/kg/day of Se for 5 weeks and 10 mg/kg/day of Aroclor 1254 in corn oil for the last 15 days; ASeD – Se-deficient group receiving Aroclor 1254 as described above.; ASeS – Se-supplemented group receiving Aroclor 1254 as described above; Control – group receiving 0.15 mg/kg/day of Se for 5 weeks; SeD – Se-deficient group receiving 0.50 mg/kg/day of Se for 5 weeks; SeS – Se-supplemented group receiving 1 mg selenium/kg/day for 5 weeks

Overall, A1254 exposure significantly impaired sperm production, motility, and morphology. Selenium supplementation partially alleviated these adverse effects. Figure 5 shows representative sperm images, and Table 1 sperm morphologies across the study groups The highest percentage of abnormal sperms was in the ASeD group, followed by the A1254, SeD, ASeS, control, and SeS groups. The predominant abnormality observed in the A1254 group was curved tail morphology, whereas the ASeD group exhibited substantial increases in both curved-tail spermatozoa and headless spermatozoa. The SeD group had increased incidence of curved mid-piece abnormalities, a finding not observed in the Aroclor 1254-treated groups. Microscopic evaluation confirmed these findings, demonstrating normal sperm morphology in the Control and SeS groups, while spermatozoa from the A, SeD, and especially ASeD groups displayed pronounced structural abnormalities, including head loss, tail curvature, coiled tails, and rudimentary tails.

**Table 1:** Sperm morphologies across the study groups

	**Control**	**SeS**	**SeD**	**A**	**ASeS**	**ASeD**
Normal sperm (%)	71.43	82.69	48.40	28.06	54.65	26.91
Sperms without tail (%)	1.75	0.43	0.99	2.36	0.96	3.79
Sperms without head (%)	5.75	4.30	8.13	12.60	10.39	23.64
Sperms with rudimentary tail (%)	0.48	0.00	0.37	1.84	1.58	3.92
Sperms with curved tail (%)	14.37	6.13	10.96	46.49	25.62	34.49
Sperms with bend mid-piece (%)	0.87	1.94	2.71	1.18	1.96	3.21
Sperms with coiled tail (%)	5.24	1.83	5.30	7.48	4.84	4.05
Sperms with curved mid-piece (%)	0.12	2.69	23.15	0.00	0.00	0.00
Sperms with swapped tail (%)	0.00	0.00	0.00	0.00	0.00	0.00
Total N of abnormal sperm (%)	**28.57**	**17.31**	**51.60**	**59.95**	**45.35**	**73.09**
Total N of sperm (%)	**100**	**100**	**100**	**100**	**100**	**100**

A – Aroclor 1254 group receiving 0.15 mg/kg/day of Se for 5 weeks and 10 mg/kg/day of Aroclor 1254 in corn oil for the last 15 days; ASeD – Se-deficient group receiving Aroclor 1254 as described above; ASeS – Se-supplemented group receiving Aroclor 1254 as described above; Control – group receiving 0.15 mg/kg/day of Se for 5 weeks; SeD – Se-deficient group receiving 0.50 mg/kg/day of Se for 5 weeks; SeS – Se-supplemented group receiving 1 mg selenium/kg/day for 5 weeks

**Figure 6:**
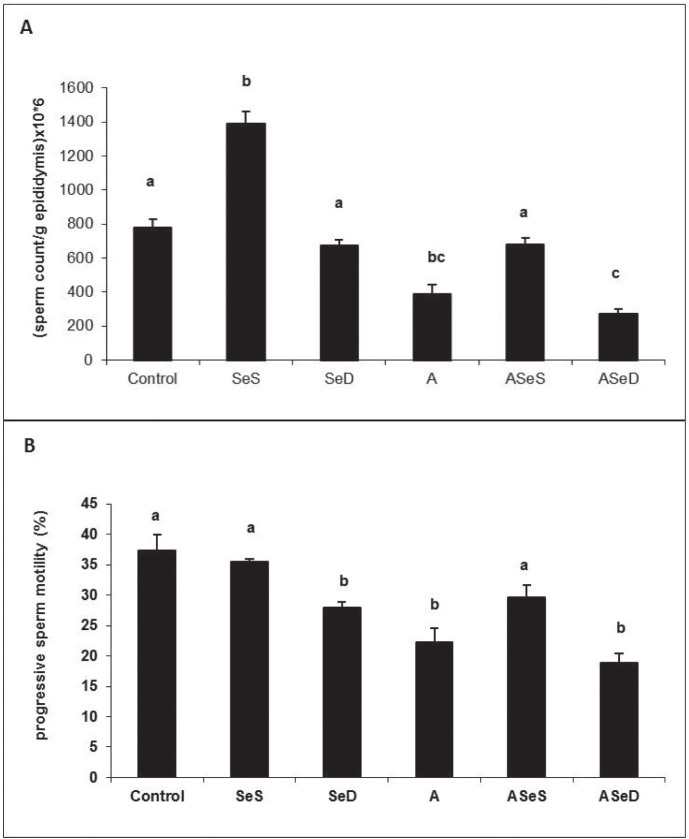
Mean sperm count (A) and viability (B) across the study groups. **^a, b, c^** Bars that do not share same superscript letters are significantly different (p<0.05). A – Aroclor 1254 group receiving 0.15 mg/kg/day of Se for 5 weeks and 10 mg/kg/day of Aroclor 1254 in corn oil for the last 15 days; ASeD – Se-deficient group receiving Aroclor 1254 as described above.; ASeS – Se-supplemented group receiving Aroclor 1254 as described above; Control – group receiving 0.15 mg/kg/day of Se for 5 weeks; SeD – Se-deficient group receiving 0.50 mg/kg/day of Se for 5 weeks; SeS – Se-supplemented group receiving 1 mg selenium/kg/day for 5 weeks

**Figure 7:**
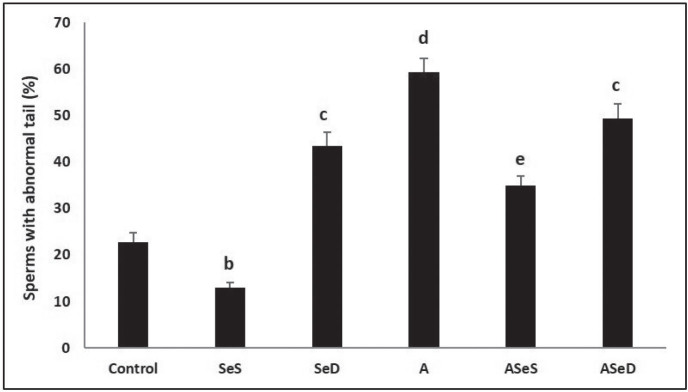
Mean prevalence of sperms with abnormal tails (%) across the study groups. **^a, b, c, d^** Bars that do not share same superscript letters are significantly different (p<0.05). A – Aroclor 1254 group receiving 0.15 mg/kg/day of Se for 5 weeks and 10 mg/kg/day of Aroclor 1254 in corn oil for the last 15 days; ASeD – Se-deficient group receiving Aroclor 1254 as described above.; ASeS – Se-supplemented group receiving Aroclor 1254 as described above; Control – group receiving 0.15 mg/kg/day of Se for 5 weeks; SeD – Se-deficient group receiving 0.50 mg/kg/day of Se for 5 weeks; SeS – Se-supplemented group receiving 1 mg selenium/kg/day for 5 weeks

Figure 6 shows sperm count and motility and Figure 7 the mean prevalence of sperms with abnormal tails. Sperm motility was significantly reduced in the SeD, A1254, and ASeD groups compared to control (p<0.05), whereas the motility in the ASeS group was comparable to the control and SeS groups, suggesting a partial protective effect of Se.

### Sex hormone levels, antioxidant enzyme activities, and oxidative stress parameters

**Figure 8:**
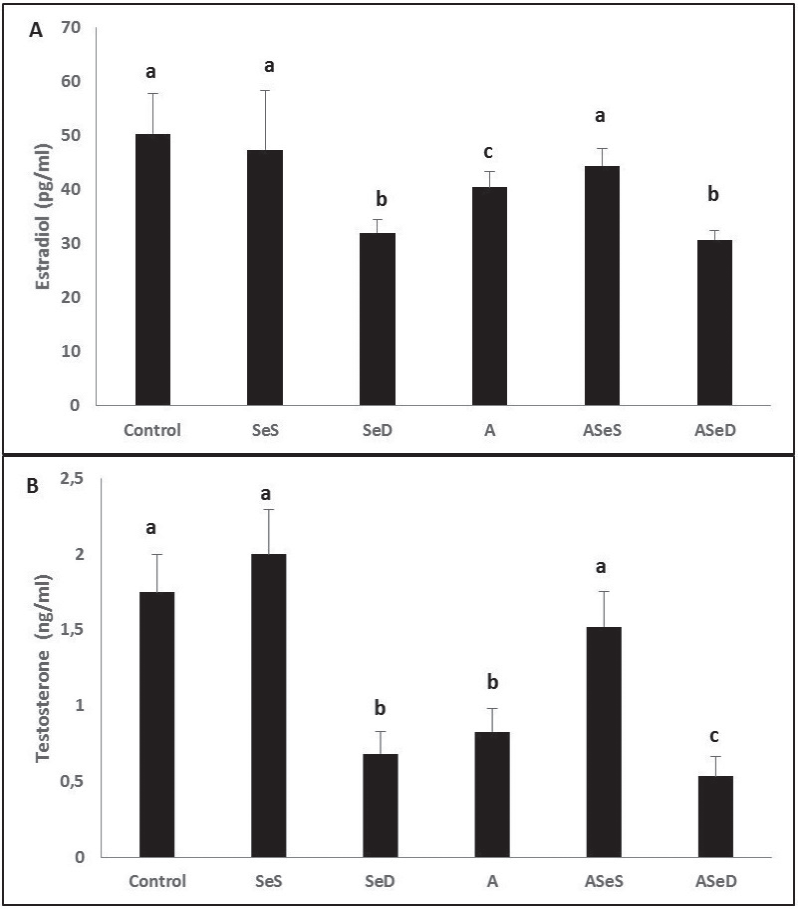
Mean oestradiol (A) and testosterone (B) levels across the study groups. **^a, b, c^** Bars that do not share same superscript letters are significantly different (p<0.05). A – Aroclor 1254 group receiving 0.15 mg/kg/day of Se for 5 weeks and 10 mg/kg/day of Aroclor 1254 in corn oil for the last 15 days; ASeD – Se-deficient group receiving Aroclor 1254 as described above.; ASeS – Se-supplemented group receiving Aroclor 1254 as described above; Control – group receiving 0.15 mg/kg/day of Se for 5 weeks; SeD – Se-deficient group receiving 0.50 mg/kg/day of Se for 5 weeks; SeS – Se-supplemented group receiving 1 mg selenium/kg/day for 5 weeks

Figure 8 shows a corresponding drop in oestradiol and testosterone levels in the Se-deficient and A1254-treated groups compared to the control (p<0.05). A similar pattern is revealed by antioxidant enzyme (GPx1, CAT, SOD, and TrxR) activities, which dropped significantly in the Se-deficient and A1254-treated groups (Table 2).

**Table 2:** Antioxidant enzyme activities; antioxidant, lipid peroxidation, protein oxidation, and selenoprotein P levels and total antioxidant capacity in the study groups

	**GPx1 (U/mg protein)**	**TrxR (U/mg protein)**	**SOD (U/mg protein)**	**CAT (nmol/min/mg protein)**	**GSH (nmol/mg protein)**	**MDA (nmol/mg protein)**	**Carbonyl groups (nmol/mg protein)**	**TAOC (nmol/mg protein)**	**SePP (ng/µg protein)**
Control	0.12±0.01**^a^**	0.19±0.02**^a^**	0.13±0.01**^a^**	3.71±0.22 **^ac^**	3.32±0.24**^a^**	1.42±0.04**^a^**	0.72±0.04**^a^**	25.27±0.60**^a^**	7.25±0.64**^a^**
SeS	0.14±0.01**^a^**	0.25±0.01**^b^**	0.14±0.02**^a^**	4.04±0.26**^a^**	3.67±0.14**^a^**	1.34±0.15**^a^**	0.63±0.08**^a^**	26.69±1.46**^a^**	8.59±1.65**^a^**
SeD	0.06±0.01**^b^**	0.12±0.02**^c^**	0.11±0.01**^a^**	2.97±0.15**^c^**	1.75±0.04**^b^**	1.55±0.07**^a^**	0.75±0.04**^a^**	26.15±0.53**^a^**	4.31±0.74**^b^**
A	0.10± 0.01**^c^**	0.15±0.02**^c^**	0.10±0.02**^b^**	1.95±0.59**^b^**	1.21±0.11**^c^**	1.95±0.09**^b^**	0.93±0.09**^b^**	24.52±0.66**^a^**	4.10±1.04**^b^**
ASeS	0.13±0.01**^a^**	0.19±0.02**^a^**	0.12±0.01**^a^**	3.75±0.2**^ac^**	3.31±0.10**^a^**	1.61±0.05**^a^**	0.83±0.08**^a^**	25.21±0.35**^a^**	4.24±1.08**^b^**
ASeD	0.08±0.02**^d^**	0.11±0.02**^c^**	0.09±0.01**^b^**	2.10±0.19**^bc^**	1.06±0.04**^c^**	2.01±0.07**^b^**	0.97±0.03**^b^**	20.93±1.68**^b^**	3.62±0.40**^b^**

a, b, c, d Bars that do not share same superscript letters are significantly different (p<0.05). A – Aroclor 1254 group receiving 0.15 mg/kg/day of Se for 5 weeks and 10 mg/kg/day of Aroclor 1254 in corn oil for the last 15 days; ASeD – Se-deficient group receiving Aroclor 1254 as described above; ASeS – Se-supplemented group receiving Aroclor 1254 as described above; CAT – Catalase; Control – group receiving 0.15 mg/kg/day of Se for 5 weeks; GPx1 – Glutathione peroxidase 1; GSH – Glutathione; MDA – Malondialdehyde; SeD – Se-deficient group receiving 0.50 mg/kg/day of Se for 5 weeks; SePP – Selenoprotein P; SeS – Se-supplemented group receiving 1 mg selenium/kg/day for 5 weeks; SOD – Superoxide dismutase; TAOC – Total antioxidant capacity; TrxR – Thioredoxin reductase

Lipid peroxidation and protein oxidation significantly increased, while TAOC and SePP levels dropped compared to control and SeS groups (p<0.05).

### Caspase-3 and 8 activities and p21 and p53 protein expressions

Reflecting these changes, caspase-3 activities were significantly higher in the A1254 and ASeD groups, whereas caspase-8 activity was significantly higher in the SeD and ASeD groups compared to control and other study groups (p<0.05) (Table 3).

**Table 3:** Caspase-3 and caspase-8 activities across the study groups

	**Caspase-3 (µmol p-NA/min/mg protein)**	**Caspase-8 (nmol p-NA/min/mg protein)**
Control	23.57±0.18**^a^**	9.21±0.01**^a^**
SeS	17.51±0.68**^a^**	9.25±0.01**^a^**
SeD	25.29±0.11**^a^**	11.65±0.01**^b^**
A	31.74±1.98**^b^**	9.17±0.23**^a^**
ASeS	22.18±1.31**^a^**	8.96±0.01**^a^**
ASeD	33.48±1.48**^b^**	12.04±0.20**^b^**

a, b Bars that do not share same superscript letters are significantly different (p<0.05). A – Aroclor 1254 group receiving 0.15 mg/kg/day of Se for 5 weeks and 10 mg/kg/day of Aroclor 1254 in corn oil for the last 15 days; ASeD – Se-deficient group receiving Aroclor 1254 as described above.; ASeS – Se-supplemented group receiving Aroclor 1254 as described above; Control – group receiving 0.15 mg/kg/day of Se for 5 weeks; p-NA – paranitroaniline; SeD – Se-deficient group receiving 0.50 mg/kg/day of Se for 5 weeks; SeS – Se-supplemented group receiving 1 mg selenium/kg/day for 5 weeks

**Figure 9:**
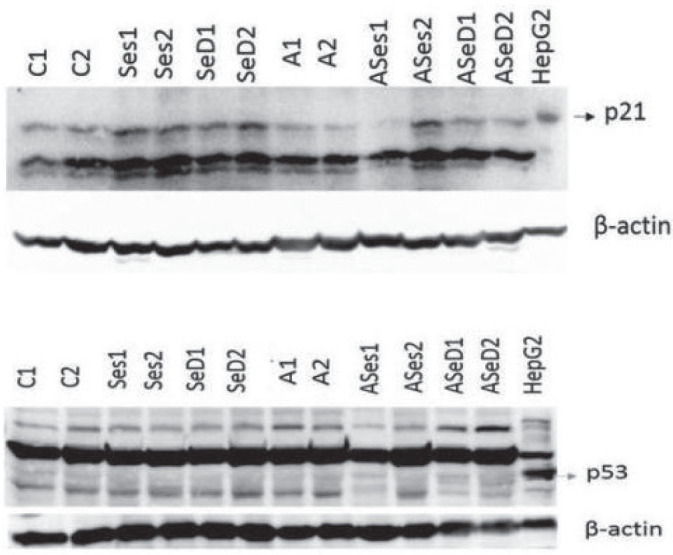
Western blot for two samples of p21 and p53 proteins for each study group. A – Aroclor 1254 group receiving 0.15 mg/kg/day of Se for 5 weeks and 10 mg/kg/day of Aroclor 1254 in corn oil for the last 15 days; ASeD – Se-deficient group receiving Aroclor 1254 as described above.; ASeS – Se-supplemented group receiving Aroclor 1254 as described above; Control – group receiving 0.15 mg/kg/day of Se for 5 weeks; HepG2 – positive control cells; SeD – Se-deficient group receiving 0.50 mg/kg/day of Se for 5 weeks; SeS – Se-supplemented group receiving 1 mg selenium/kg/day for 5 weeks

In contrast, we found no significant difference in p21 and p53 expressions across all groups (Figure 9).

## DISCUSSION

Our findings confirm the adverse effects of A1254 exposure on testicular function and parameters and point to oxidative stress as its primary driver, but more importantly, they reinforce our hypothesis that Se supplementation would provide partial protection against A1254 toxicity, most likely by boosting antioxidative action to maintain the redox status and membrane integrity, as reported by many studies (21,22,23,24,25,26,27,28,29).

Mechanistically, we have also confirmed that A1254 induces apoptosis primarily via the caspase-3 (intrinsic) and caspase-8 (extrinsic) pathways as reported earlier (30). Consistent with its protective role as an important trace element, Se countered these effects. However, we found no significant differences in p53 and p21 protein expressions across the experimental groups, which is surprising, considering that p53 and p21 are the key cell-cycle checkpoint proteins involved in mediating apoptosis and cell cycle arrest (31). This discrepancy may be owed to the specific dose and exposure duration (15 days), which may have been insufficient to trigger significant protein transcription or translation in the testis tissue.

### Study limitations

The interpretation of our findings may be limited by the use of a single A1254 dose over a short, 15-day period, which, while effective for demonstrating acute toxicity, may not fully reflect chronic low-level exposure common in environmental settings. Furthermore, our study employed a fixed supplementation dose of Se, whereas a dose-response analysis would provide stronger evidence for defining an optimal therapeutic concentration. Finally, while we investigated protein expression (p53 and p21), the study did not include direct analysis of enzyme kinetics or receptor binding assays, which limits our conclusion about the mechanistic pathways in every instance.

## CONCLUSION

Limitations aside, our findings are novel in demonstrating that Se supplementation can significantly mitigate the adverse effects of PCBs by restoring redox homeostasis, reducing apoptosis, and normalising sperm and hormone parameters. Nevertheless, Se alone may not be sufficient to completely overcome the reproductive toxicity associated with persistent environmental contaminants such as A1254.
